# Sequential Production of Sodium Alginate and Biomethane from Holopelagic *Sargassum* spp. to Promote a Circular Economy in the Mexican Caribbean

**DOI:** 10.3390/md24080292

**Published:** 2026-08-21

**Authors:** Karla J. Azcorra-May, Elda I. España-Gamboa, Liliana Alzate-Gaviria, Jorge A. Domínguez-Maldonado, Tanit Toledano-Thompson, Rosa M. Leal-Bautista, José M. Cervantes-Uc, Raúl Tapia-Tussell

**Affiliations:** 1 Renewable Energy Unit, Centro de Investigacion Cientifica de Yucatan, Merida CP 97205, Mexico; karlaazcorra1@gmail.com (K.J.A.-M.); elda.espana@cicy.mx (E.I.E.-G.); lag@cicy.mx (L.A.-G.); joe2@cicy.mx (J.A.D.-M.); tanit@cicy.mx (T.T.-T.); 2 Water Unit, Centro de Investigacion Cientifica de Yucatan, Cancun CP 77524, Mexico; rleal@cicy.mx; 3 Materials Unit, Centro de Investigacion Cientifica de Yucatan, Merida CP 97205, Mexico; manceruc@cicy.mx

**Keywords:** biorefinery, seaweed, bioproducts, biofuels, sustainable valorization

## Abstract

This research proposes an approach based on a circular economy principle for the integral valorization of *Sargassum* from the Mexican Caribbean. The biomass was characterized through proximal and elemental analyses, and then an oxidative pretreatment was carried out to enhance a sequential processing scheme to extract sodium alginate and use the solid waste as a substrate for biogas production via anaerobic digestion. The oxidative pretreatment successfully reduces the recalcitrant content and the concentration of heavy metals. The sodium alginate extracted from treated biomass achieves a yield higher than 20%; the characterization of the polymer via nuclear magnetic resonance showed that the mannuronic-to-guluronic ratio was between 0.34 and 0.62, indicating the potential for its use for environmental and biomedical applications. The highest yield in methane production was 328 mL CH_4_/g of volatile solids, with a purity of 90%, and was achieved using the waste from alginate extraction with an inoculum-to-substrate ratio of 1:1. The experimental data presented an excellent fit to a Gompertz model (R^2^ > 0.99). The proposed valorization pathway improves the sustainability of *Sargassum* management, prioritizing the recovery of high-value compounds before energy production. This circular approach provides a framework for converting environmental challenges into opportunities in the Caribbean.

## 1. Introduction

Over the last decade, massive amounts of *Sargassum* from the Great Atlantic *Sargassum* Belt (GASB) have reached the Caribbean, creating an environmental and socioeconomic crisis [1]. This phenomenon disrupts the ecological balance of the ecosystem, affecting marine species such as fish, coral reefs and turtles [2]; its accumulation blocks sunlight, creating anoxic conditions that generate negative impacts on different species. Then, when they reach the coast, a decomposition process starts that produces noxious gases and releases leachates containing high amounts of heavy metals [3].

Despite these negative impacts, *Sargassum* contains valuable compounds such as alginates, fucoidan, glucose and xylose, as well as polyphenols, which can be used as a feedstock to obtain bioproducts and biofuels under a sustainable valorization approach [4].

The industrial exploitation of *Sargassum* has been limited due to the complexity of its lignocellulosic matrix and the presence of heavy metals that compromise the application of the compounds produced from the seaweed [5]. Several authors, such as Milledge et al. [6], mentioned that high levels of phenolic compounds might affect the anaerobic digestion process by decreasing methane production yield. Considering that, different pretreatments have been explored to increase bioproduct recovery yields; hydrothermal treatments are a promising technology for bioethanol and biogas production [7,8,9,10,11,12]. Thompson et al. [10] achieved an increase in methane yield using a hydrothermal pretreatment, raising from 41.84 to 116.72 mL CH_4_/gVS. Conversely, Aparicio et al. [12] use a high-pressure technology and an enzymatic saccharification to achieve an ethanol conversion yield of 76.23%; these treatments increase the potential of the biomass for its exploitation; however, they use harsh conditions and have high energy and water requirements, highlighting the need to develop scalable pretreatments with low energy requirements that modify the structure of macroalgae and reduce metal concentration.

Besides the technical advantages of pretreatments, it is important to assess the environmental feasibility of the process to ensure its sustainability [13]. New trends are encouraging the transition from a linear to a circular economy by adopting integrated biorefinery approaches that maximize the exploitation of the feedstock and promote zero-waste generation [14,15,16,17].

In a previous study [7], an oxidative pretreatment was successfully developed to remove the 70% of lignin-like compounds, thus enhancing access to carbohydrates and the biotransformation process. Furthermore, a life cycle assessment (LCA) of the pretreatment was conducted to analyze its sustainability; the results indicated that the oxidative pretreatment presented a more favorable sustainability profile compared to conventional methods [18]. While these findings support the environmental viability of the process, and considering its potential to enhance valorization, further research is necessary to establish its technoeconomic feasibility for large-scale implementation.

This study proposes a comprehensive valorization pathway that includes an oxidative pretreatment, followed by a sequential sodium alginate extraction and the subsequent conversion of the solid residue into biomethane through anaerobic digestion; the main contribution relies on the development of a scalable framework for the sustainable management of *Sargassum*.

## 2. Results and Discussion

### 2.1. Sargassum Biomass (SB) Characterization

The proximal analysis of the *Sargassum* biomass (SB) is presented in Table 1. The ash content had a low value (16.36 ± 0.21%) compared with other seaweeds collected in the Caribbean and can be related with the presence of lower amounts of salts, minerals, and metals. Such variations are likely driven by the specific collection site and seasonal dynamics, as noted by Vazquez-Delfin et al. [19], who reported ash values between 18.10 and 23.90% for *Sargassum* collected across various locations in Quintana Roo, Mexico. Also, Milledge et al. [6] and Thompson et al. [10] reported notably higher values of ashes for *Sargassum* collected at Turks and Caicos Islands (31.82%) and Barbados (46.94%), respectively, during the massive influxes in 2019.

Although lignin is characteristic of vascular plants rather than marine algae, recent studies indicate lignin-like compound values up to 20% [4,5,20,21]. These reports are consistent with the findings in this work, in which lignin-like content was 26.43 ± 1.75. The presence of high amounts of a recalcitrant material can be related to the findings of Martone et al. [22], who discovered that the red seaweed *Calliarthron cheilosporioides* has a secondary cell wall, as well as derived compounds from *p*-hydroxyphenyl (H) and guaiacyl (G) units, produced due to major evolutionary process as a response to mechanical stress.

There are also reports of a secondary cell wall in *Sargassum* samples as well as the presence of *p*-coumaric acid, a precursor of monolignol units, that indicates that brown seaweed from the Caribbean had a recalcitrant lignin-like compound [4]. The presence of lignified cells and precursors for monolignol biosynthesis explains why these values exceed those of most macroalgae but remain consistent with biomass collected in the Caribbean [5]. It is important to point out that the high recalcitrance (exceeding 20%) reduces the proportion of available carbohydrates, suggesting that pretreatments are essential to access the valuable compounds and unlock the biomass’s full potential [23].

The carbohydrate profile showed glucose at 6.05 ± 0.03%, xylose at 1.71 ± 0.01%, and fucose at 1.10 ± 0.31%, while glucose and xylose are precursors for bioethanol and biogas production through their association with cellulose and hemicellulose; the simultaneous presence of fucose and sulfur (0.78 ± 0.03%) highlights the potential for recovering high-value sulfated fucoidans with bioactive properties [24,25].

Finally, the elemental analysis showed carbon and nitrogen contents of 37.62 and 0.98%, respectively, resulting in a C:N ratio of 38.38. This ratio is important for biotransformation processes like anaerobic digestion, where nitrogen deficiency can lead to partial or total process inhibition. Additionally, the sulfur level is relevant for biogas production since it can be transformed and released as hydrogen sulfide (H_2_S) [26].

### 2.2. Evaluation of the Oxidative Pretreatment

As Table 1 shows, the oxidative pretreatment modifies the biochemical composition of *Sargassum*. One of the most relevant results was the significant reduction in the recalcitrant material (lignin-like compound), which decreased from 26.43% to 7.60%. This confirms the effectiveness of the oxidative pretreatment in reducing the recalcitrant content and breaking down the phenolic matrix. Milledge et al. [6] mentioned that the high phenolic content in the *Sargassum* can lead to the inhibition of microorganisms in anaerobic digestion, since it may interfere with the hydrolysis of complex molecules in the early stages of biomethane production. On the other hand, the HPLC analysis showed an increase in the concentration of carbohydrate monomers; glucose levels increased from 6.05% to nearly 9.0%, while xylose and fucose also showed upward trends in the treated biomass. This enrichment is likely due to the remove of lignin-like compounds, which increased the potential and accessibility of carbohydrates.

Although that the oxidative treatment used in this work is a novel approach that is currently under patent application (MX/a/2023/014346), there are other chemical process applied in different feedstock yielding lignin removal rates between 5 and 33% using an enzymatic process in sugarcane bagasse and around 40 to 88% using a combine process with a thermochemical treatment and two acids (acetic and nitric) [27,28].

Regarding the elemental analysis, the carbon (C), hydrogen (H), and sulfur (S) concentrations remained consistent after treatment, with a slight increase in nitrogen (N). This stability in the elemental concentration, combined with the successful delignification and carbohydrate release, demonstrates that oxidative pretreatment is a highly efficient strategy for enhancing the valorization potential of the feedstock. By lowering the lignin-to-carbohydrate ratio, the biomass becomes significantly more viable for high-yield biofuel production and other biorefinery applications [7].

### 2.3. Alginate Yield and Characterization

The alginate yield from untreated biomass (SB) was 35.00 ± 1.32%, while the treated *Sargassum* (TS) decreased the value, obtaining a recovery of 27.90 ± 1.78% on a dry basis. Although oxidative treatment decreased the alginate yield, both values exceed 20%, which is an attractive value for industrial processing [29,30]. Is also important to consider other parameters besides the yields that determine the quality of the alginate.

The yields are consistent with those reported for other species, such as *S. wightii* (31.70%) and *S. polycystum* (30.17%) [31,32]. However, they are higher than the range of 17–23% reported for *S. natans* and *S. fluitans* from the Caribbean [33,34]. These results indicate that pelagic *Sargassum* from the Caribbean can be a promising feedstock for alginate extraction.

The decrease in the alginate yield after the oxidative pretreatment can be due the cleavage of glycosidic bonds (C-O-C) in the acid alginic, these bonds are responsible to keep joint the mannuronic and guluronic acid monomers [35]; in the pretreatment hydrogen peroxide is used as an oxidizing agent to break β-O-4 ether bonds from the lignin; however, it can target phenolic and non-phenolic structures [36]. A previous study using *Laminaria digitata* successfully depolymerizes sodium alginate by using hydrogen peroxide; this process can be used to obtain alginate oligosaccharides, which have potential applications as additives [37].

Even if the alginate from SB and TS is above the yield considered viable for industrial exploitation, it is important to consider not only the yield, but also the properties of the compound to determine potential applications. In this study, besides the yield, the functional groups, structural compound proportions (mannuronic and guluronic acid) and metal concentration in alginate were also determined.

**Figure 1:**
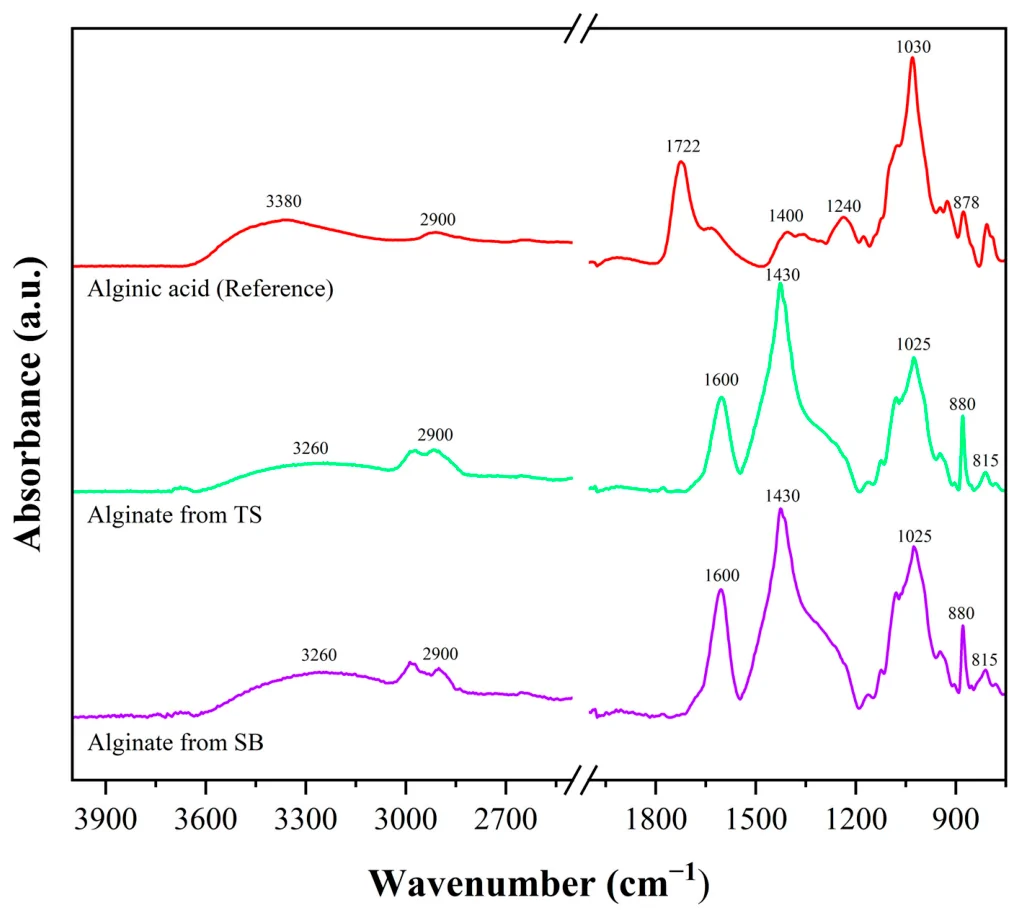
Fourier transform Infrared (FT-IR) spectra of the alginate from *Sargassum* biomass (SB), treated *Sargassum* (TS) and alginic acid.

FT-IR spectra from the treated and untreated *Sargassum*, as well as the reference compound (alginic acid), were presented in Figure 1, and it was used to confirm the successful conversion of alginic acid into sodium alginate and to evaluate the structural differences produced by the oxidative pretreatment in the alginate. A major difference between sodium alginate and alginic acid was the absence of the 1722 cm^−1^ band in the extracted samples, and the presence of a signal at 1600 cm^−1^. This agrees with the reports of El Atouani et al. [38], who mentioned that the stretching of C=O from carboxylic acid appears around 1700 cm^−1^ and is characteristics to alginic acid, while at 1600 cm^−1^ the signals of the asymmetric bonds in O-C-O from the sodium alginate carboxylate anions are present. The lack of the band at 1700 cm^−1^ in the extracted alginate indicates the full conversion of alginic acid into sodium alginate.

The presence of the pyranose ring in all samples was confirmed by the C-O-C stretching at 1025 cm^−1^ [39] and is associated with the identification of carbohydrates. Furthermore, the fingerprint region (950 and 750 cm^−1^) exhibited signals at 880 and 815 cm^−1^, which were attributed to the C1-H deformation of mannuronic acid [38]. The spectral similarity between the SB and TS samples suggests that oxidative treatment does not significantly degrade the polymer’s primary functional groups.

Meanwhile, the structural composition of the samples was elucidated by ^1^H-NMR spectroscopy. Table 2 shows that both SB and TS had a higher proportion of guluronic acid (G) than the commercial reference. These results suggest that the oxidative pretreatment has an impact on the resulting alginate by promoting the loss of mannuronic acid fractions.

It is important to determine the mannuronic-to-guluronic (M/G) ratio since it is directly related to the viscosity, emulsifying capacity and antioxidant activity of alginates. A high proportion of guluronic acid improves bioactive properties; thus, a low M/G ratio can be associated with a high bioactivity [40].

The low M/G ratios observed in the samples are consistent with those reported by Rhein-Knudsen et al. [34] for *S. natans* from Ghana, where the value was 0.47. However, it is important to point out that other species of *Sargassum*, such as *S. muticum*, *polycystum* and *filipendula*, usually have a balanced proportion of F_G_ and F_M_, with ratios close to 1 [41,42], indicating that pelagic *Sargassum* from the Caribbean (*S. fluitans* and *natans*) tends to have a higher proportion of guluronic acid and a lower M/G ratio.

Alginates with low M/G ratios, such as the one obtained from pelagic *Sargassum*, are associated with the formation of rigid, thermostable gels, since guluronic blocks selectively chelate divalent cations through an “egg-box” mechanism that creates ordered linkage zones, providing stronger unions compared with alginates with high mannuronic content [43]. This property makes alginates with low M/G ratio ideal for cell encapsulation, drug delivery systems and environmental remediation [44]. Consequently, alginates with rich G-blocks are ideal for biomedical and environmental applications, while M-rich alginates create more elastic and fluid gels that are required for the food industry [45].

Structural property is a key factor that indicates the most suitable application for the alginate; however, it is necessary to evaluate other properties, such as purity, molecular weight, viscosity, biocompatibility, and so on. Therefore, while the low M/G ratio in *Sargassum* suggests potential for biomedical applications, this study is the first step to valorize the biomass, and further purification and characterization studies are necessary to define specific applications.

### 2.4. Trace Metal Analysis

Metal concentration in *Sargassum* can vary according to the season, harvesting point and species within the biomass. The presence of heavy metals such as arsenic, aluminum, and cadmium in the seaweed limits its applications, considering that the analysis and the study of traceability can allow the establishment of quality and safety criteria for the valorization of *Sargassum*.

**Figure 2:**
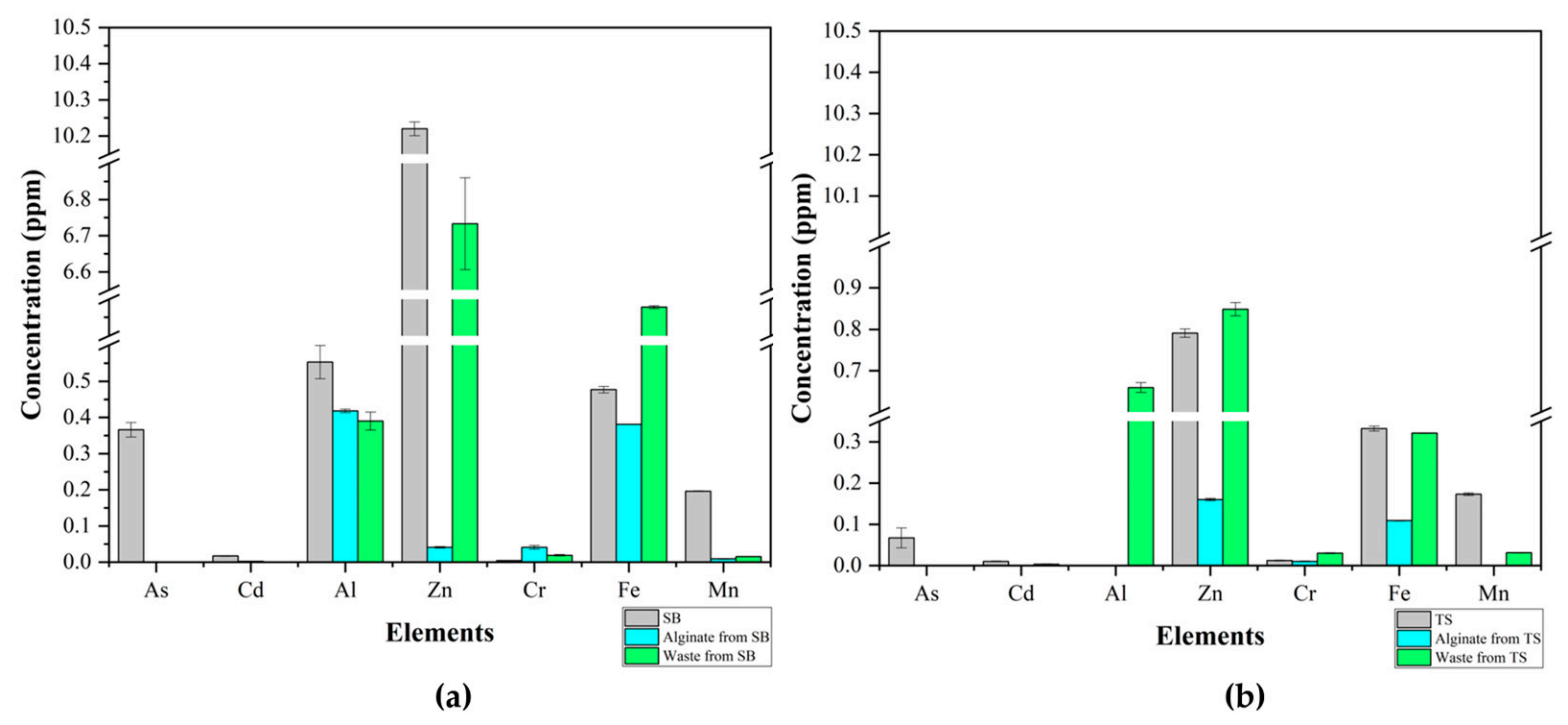
Metal traceability during the alginate extraction process using (**a**) *Sargassum* biomass (SB) and (**b**) treated *Sargassum* (TS).

Figure 2 shows the presence of metals during the alginate extraction process for SB and TS. In general, a significant decrease in the concentration of all analyzed metals was observed because of the oxidative pretreatment.

In SB, zinc reached a concentration of 10.2 ppm, although this value remains below the maximum permissible limits for biosolid disposal established by the Mexican regulation NOM-004-SEMARNAT [46]. The oxidative process remarkably reduced Zn levels by 90%; this reduction highlights the oxidative treatment’s efficiency in detoxifying the biomass. In high concentrations, Zn can promote phytotoxic effects due to its ability to be absorbed by plants [47]. On the other hand, aluminum in high concentrations may cause negative impacts on marine ecosystems by polluting water resources and damaging the nervous system of marine organisms, which can access the food chain and promote neurodegenerative diseases, as well as liver and kidney damage; besides the impacts on marine ecosystems, Al can also have phytotoxic effects, inhibiting plant growth and germination rates [48].

*Sargassum* species usually contain arsenic in their composition [5,6,49,50,51,52], this element is one of the most dangerous metals for human health, and there have been several reports of poisonings in China, Bangladesh and in mineral areas in Mexico. Even though the metal concentration in the biomass was initially low and below the Mexican regulations (NOM-004-SEMARNAT-2002) for the use and final disposal of sludge and biosolids [46], it is important to monitor the concentration of the elements, since their high concentration can limit the applications of the value-added compounds obtained from *Sargassum* [53].

An analysis of metal distribution during the alginate extraction process shows that the metals remain in the solid waste from the process (W), which is typically considered as a waste, producing that the alginate showing a lower metal concentrations than the initial biomass. It is important to highlight that the alginate has less metal content than W. However, when the pretreatment is used, even the metal concentration in W is lower than the maximum permissible limits for discharging wastewater into water sources (NOM-001-SEMARNAT-2021) [54].

It is important to highlight that the alginate present has less metal content than W. However, when the pretreatment is used, even the metal concentration in W is lower than the maximum permissible limits for wastewater discharges into receiving bodies.

The low affinity of the target biopolymer for these metallic ions during the alkaline extraction phase is a major technological advantage, since it allows the extraction of higher purity compounds. The decrease in metals indicates that they are likely bound to the carboxyl groups of the lignin-like and are precipitated during pH shifts in the alginate extraction process [55,56]. Considering the properties of the alginate and that the oxidative treatment allows the decrease in metal content, the residual biomass from the TS process was selected as feedstock for biogas production.

### 2.5. Biomethane Potential of the Waste from the Alginate Extraction

Volatile solids (VS) in the inoculum were 13.54 g/L, this parameter was used to adjust the inoculum-to-substrate (I:S) ratio, the waste from the alginate extraction process using TS was named as W and it was used as a feedstock for anaerobic digestion, the treated *Sargassum* was used as a control (TS) to evaluate the biomethane production, also a blank compose only by the inoculum was used and represented as Blk.

**Figure 3:**
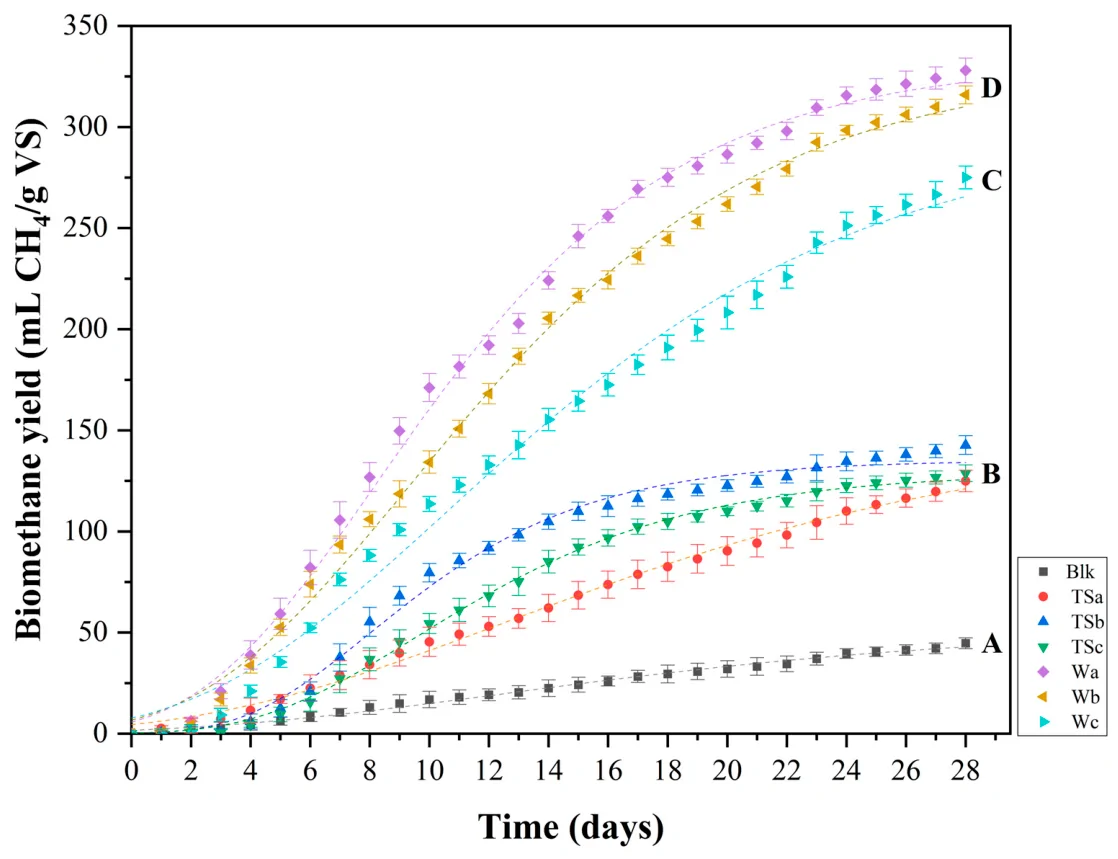
Cumulative methane yield (mL CH_4_/gVS) of the blank (Blk), treated *Sargassum* (TS) and the waste from the alginate extraction process using treated *Sargassum* (W) with different inoculum to substrate ratio (I:S) a = 2:1, b = 1:1 and c = 1:2. Different letters (A, B, C and D) represented a statistical difference (*p* < 0.05).

Cumulative methane yields over 28 days are presented in Figure 3; they followed a nonlinear sigmoidal pattern characterized by three growth phases: an adaptation or lag phase with slow initial growth, followed by an exponential increase and system stabilization [57]. This pattern was accurately described by the modified Gompertz model (R^2^ > 0.99). As expected, the Blk exhibited the lowest methane yields; the yields reported in Figure 3 were adjusted by subtracting the production generated by the blank. W achieved higher yields compared to ST, reaching over 320 mL CH_4_/gVS using an I:S of 2:1 and 1:1; meanwhile, ST achieved less than 150 mL CH_4_/gVS in all treatments.

TS showed yields of 124.40–142.48 mL CH_4_/gVS, similar to results obtained from swine manure, fruit waste and other *Sargassum* species, such as *S. ilicifolium* with a methane yield of 177 mL CH_4_/gVS [58]. *Sargassum* from the Caribbean usually has low methane yields, probably due to its recalcitrant structure and the presence of inhibitors, which limit the transformation of organic molecules; Milledge et al. [6] attribute the inhibition of the microorganisms in anaerobic digestion to the high content of salt, metals and polyphenols in the *Sargassum* from Turks and Caicos. *Sargassum* collected from the Dominican Republic presented yields ranging from 79.68 to 101.30 mL CH_4_/gVS and the macroalgae from the Mexican Caribbean showed lower values within the range of 83.45–90 mL CH_4_/gVS [8,59,60].

The notable increase in methane yield using *Sargassum* from the Mexican Caribbean was due to the oxidative pretreatment, which modifies the structure of the biomass, reduces the recalcitrant material and improves the bioconversion [7]. In TS, the optimal I:S ratio was 1:1, which agrees with previous studies; Owamah [61] found that the balance in the inoculum-to-substrate ratio prevents acidification in the anaerobic digestion process; nevertheless, no statistical variations were found in TS when different I:S ratios were used.

As previously mentioned, the waste from the alginate extraction process (W) presented the highest methane yields with values of 327.80 mL CH_4_/gVS, and the shortest lag times, indicating a better biodegradability of the substrate. The yields are similar to those reported by Chikani-Cabrera et al. [62], who obtained 387 mL CH_4_/gVS using *Sargassum* with combined treatments, including chemical compounds and enzymes; the results are also comparable to those obtained from chicken and pig manure and fruit and vegetable waste, in which the methane yields are 295, 322 and 342 mL CH_4_/gVS, respectively [63].

In W, the best I:S ratios were 2:1 and 1:1, highlighting the relevance of this parameter, since it can impact the efficiency and stability of the anaerobic digestion. A high proportion of inoculum can lead to the acidification of the medium; meanwhile, a low content of inoculum can promote a slow or incomplete conversion [57]. In this case, the ratio 1:1 is the best condition for biomethane production, given that there is no statistical difference among them and a higher amount of biomass can be used. On the other hand, the treatment with higher substrate than inoculum (2:1), using W, presents a lower yield, which might be due to the presence of inhibitors that affect the process.

Besides that, the use of the waste from the alginate extraction duplicates the biomethane yield; it also benefits the valorization pathway by promoting zero-waste generation.

In Table 3, the kinetic parameters obtained from the modified Gompertz models are presented; the shorter lag phase (λ) and higher maximum production rate (μ) for W confirm that removing alginate reduces the structural recalcitrance of the seaweed, facilitating faster hydrolysis and enhancing the methane yield. All experimental data also presented an excellent model fit, with a coefficient of determination above 0.99; this shows that the model accurately represents the characteristics of the process.

The maximum production potential (A) was obtained using W, and the results were around 299.35 and 332.96 mL CH_4_/gVS; these values are significantly higher than those obtained using TS; in both samples, the I:S ratio of 1:2 presented the lowest methane yield, indicating that the ratio 1:1 is more accurate. TS and W presented a higher production potential compared with the results reported by Paletta et al. [8] and Castro et al. [59], in which the monodigestion of *Sargassum* had maximum values of 79.70 and 100 mL CH_4_/gVS, respectively. However, these are similar to the values reported for the-co digestion of *Sargassum* and food waste in the Dominican Republic, in which the results were between 327.30 and 615 mL CH_4_/gVS [59].

The lag phase in the samples lasted between 2.35 and 4.29 days; these were shorter than the ones reported by Lopez-Aguilar et al. [60], in which the monodigestion of *Sargassum* has a lag phase of 7 days. The maximum production rate (µ) indicates microbial activity; a low rate indicates low activity or inhibition, while high rates are associated with profitable activity. In this study, all samples showed differences in this parameter. However, W had exceptionally high values, demonstrating the potential of waste as a feedstock for anaerobic digestion.

**Figure 4:**
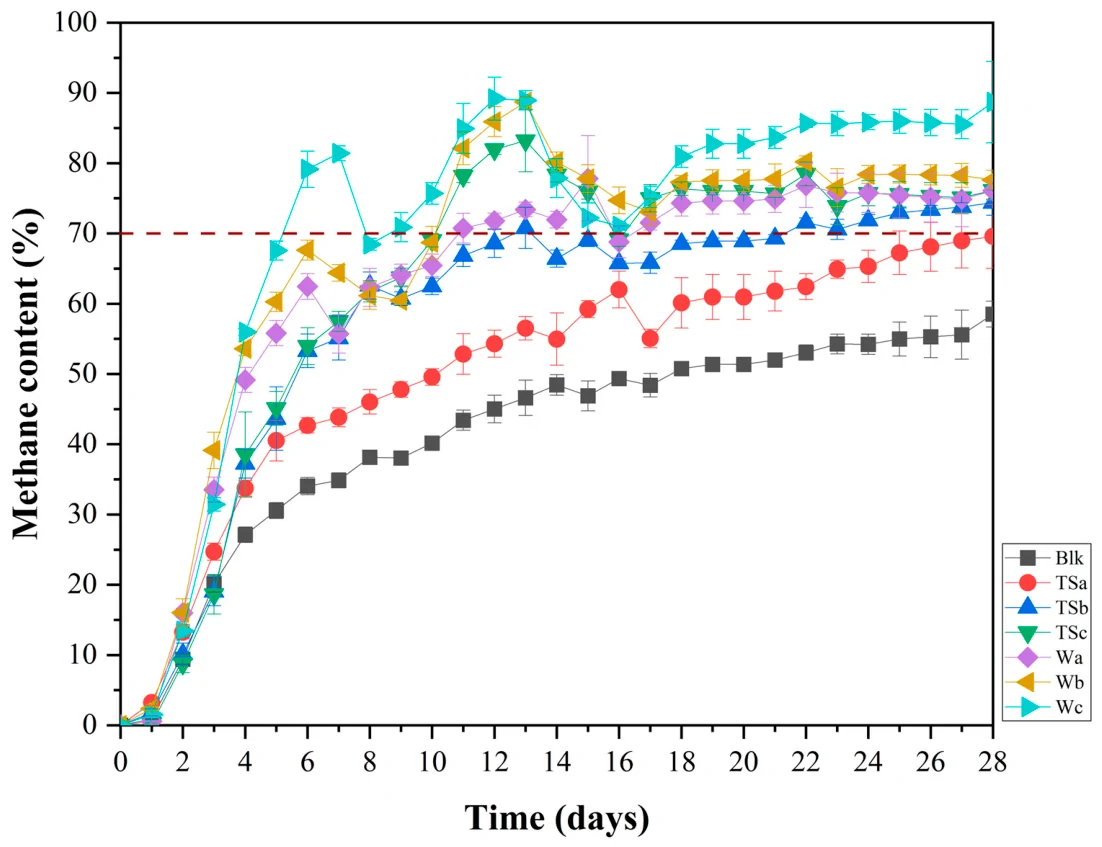
Methane content (%) in biogas during a 28-day anaerobic digestion process using different samples. Blk = blank, TS = treated *Sargassum*, W = waste from the alginate extraction process using treated *Sargassum*. The horizontal red dashed line indicates 70% methane content which is the minimum threshold value for methane in biogas.

A remarkable outcome of this research is the high methane content in the resulting biogas (Figure 4), one goal for energy applications is that the biogas has at least 70% methane in its composition [64]; W samples successfully achieve a methane content near 90% by day 13. This level of purity is notably higher than the 50–60% typically reported in the monodigestion of *Sargassum* [65]. Thompson et al. [10] successfully improved the methane yield by using a hydrothermal pretreatment at high temperatures; however, the maximum methane concentration was around 50%, similar to what was reported by Tapia-Tussell et al. [66], who reported a concentration of 52% when a biological treatment was used.

The high methane purity can be explained by the combined effect of the two processes applied prior to anaerobic digestion. The oxidative pretreatment reduces the recalcitrance of the *Sargassum* by removing polyphenols, which can inhibit methanogenic bacteria, enhancing biodegradability [6,10]. Afterwards, alginate was extracted under alkaline conditions, leaving a solid waste (W) enriched with simpler fractions that facilitate the transformation into methane. Conversely, the study conducted by Candia-Lomeli et al. [67] demonstrates that alkaline conditions enhance the biomethane potential of the microalgae *Scenedesmus obtusiusculus,* achieving a methane content up to 91%. The alkaline conditions can contribute to higher absorption in the digestion medium, improving the methane content of the biogas [68].

Regarding the TS, the I:S ratio of 2:1 is not suitable for biomethane production since the methane content in biogas is less than 70%; this means that it is necessary to add a larger amount of biomass to achieve an optimal methane concentration, which is a technical advantage in that more *Sargassum* can be used in the process.

The increase in methane purity can be due to the oxidative pretreatment combined with alginate extraction, which removes inhibitory compounds such as lignin-like compounds and complex polyphenols, resulting in a more suitable feedstock for the biotransformation process; this points out that the pretreatment not only increases the biomethane yields but also improves the quality of the bioproducts.

The theoretical biomethane potential (TBMP) was calculated from the empirical formula, and it was compared with the experimental results to obtain the biodegradability index (BI); it was significantly higher for W than for TS (Table 3), confirming that the alginate extraction waste is a more efficient feedstock for methane conversion. This enhancement of energy recovery in the remaining fraction through the extraction of one product is the hallmark of an optimized *Sargassum* biorefinery.

## 3. Materials and Methods

### 3.1. Sargassum Collection

The *Sargassum* biomass (SB) was collected at Puerto Aventuras in Quintana Roo, Mexico (20°29′49″ N, 87°13′56″ O); after the collection, the biomass was transferred to the Yucatan Science and Technology Park in Sierra Papacal, Yucatan, for processing. This process involved washing the biomass with tap water, sun-drying, milling and sieving it using a mesh size No. 20 and 40 to obtain a dried biomass within a particle size of 425–850 microns, which was stored in plastic bags for its further characterization and pretreatment [4].

### 3.2. Oxidative Pretreatment

The dried SB was subjected to an oxidative pretreatment using the methodology proposed by Azcorra et al. [7] to modify its structure and enhance its bioconversion into value-added compounds. The process was carried out using a stirred tank reactor (STR), in which 300 g of SB was added and mixed for three hours at 40 °C and 150 rpm with three liters of hydrogen peroxide at 10% *v*/*v*. Then, the solid and liquid fractions were separated by filtration; the solid fraction was named treated *Sargassum* (TS); it was washed with tap water to remove residual peroxide and then dried in a convection oven for 24 h at 70 °C. TS was characterized to evaluate the pretreatment efficiency and used as a feedstock for alginate extraction.

### 3.3. Alginate Extraction

The alginate extraction from SB and TS was achieved through the method established by Ardalan et al. [69], in which 10 g of dried biomass was subjected to an acid pre-extraction step, using 75 mL of 0.2 M hydrochloric acid (HCl); after the pre-extraction, the solid and liquid fractions were separated through filtration; and the solid fraction underwent to an alkaline hydrolysis by adding 250 mL of 2% sodium carbonate (Na_2_CO_3_). The mixture was heated to 80 °C for two hours, then filtered using a vacuum pump. The solid waste, that is, the *Sargassum* without alginate, was designated W and stored at 4 °C to use as feedstock for anaerobic digestion.

The liquid fraction contains soluble alginic acid, which was converted into soluble alginic acid and sodium alginate by adding HCl (0.2 M) and Na_2_CO_3_ (2% *w*/*v*) respectively. The sodium alginate was then precipitated with 96% *v*/*v* ethanol, dried at 45 °C for 24 h, and stored for its further characterization.

### 3.4. Biochemical Methane Potential (BMP)

As mentioned above, the waste from the alginate extraction process (W) was used as substrate for biomethane production to promote a circular economy approach; a microbial inoculum with anaerobic microorganisms was mixed with the substrate to promote anaerobic digestion.

#### 3.4.1. Inoculum Preparation and Characterization

The anaerobic microbial community was obtained from an inoculum prepared by mixing 300 g/L of cattle manure, 30 g/L of deep soil, 1.5 g/L of commercial sodium carbonate, and 5 g/L of sugar [66]. The inoculum was degassed by incubating it for five days at 38 °C. Subsequently, the concentration of total solids (TS) and volatile solids (VS) was determined following the NREL techniques [70].

#### 3.4.2. Methane Quantification and Biogas Composition

The biochemical methane potential was evaluated according to the methodology proposed by Tapia-Tussell et al. [66]. Three inoculum-to-substrate ratios were assessed (2:1, 1:1, and 1:2 g of volatile solids (VS) in the inoculum per g of VS in the substrate). Serological bottles of 250 mL were used for the test, and the substrate, inoculum, and water were added into them until they reached a volume of 150 mL, leading to a headspace of 100 mL.

The bottles were sealed with a rubber septa and metal rings. Then, nitrogen was injected into an anaerobic chamber to remove the oxygen and ensure anaerobic conditions; the bottles were incubated under static conditions at 38 °C for 28 days. The inoculum without substrate was used as a blank to adjust the yields, considering the methane produced only by the blank (Blk). All experiments were performed in triplicate.

The manometric pressure generated during the anaerobic digestion process was monitored daily using a digital pressure transducer (PN2596, ifm electronic GmbH, Essen, Germany) coupled to a syringe. The accumulated pressure within each flask was released due to its venting to restore baseline conditions. The volume of biogas produced, normalized to standard temperature and pressure (STP: 0 °C and 1 atm), was determined according to the following equation.

V_biogas_ = (V_headspace_) × (P_headspace_/P_STP_) × (T_STP_/T) (1)

where V_biogas_ represents the standardized biogas volume, while V_headspace_ corresponds to the free volume of the serum flask (100 mL). P_headspace_ denotes the manometric pressure measured in mbar, P_STP_ is the standard pressure (1013.25 mbar), T_STP_ is the standard temperature (273.15 K), and T is the operational temperature of the experiment (311.15 K).

To characterize the biogas composition, gas samples were collected every 24 h to determine methane concentration through gas chromatography using a Perkin Elmer gas chromatograph (PerkinElmer Inc., Waltham, MA, USA); the stationary phase was an Elite-MOLESIEVE GC column (30 m length, 0.53 mm internal diameter). The analytical conditions were set with injector, oven, and detector temperatures at 150, 60, and 200 °C, respectively. Nitrogen was used as the carrier gas, and the calibration curve was performed by injecting standard methane (99% purity) in volumes ranging from 250 to 750 µL.

Finally, the dry methane percentage within the biogas was adjusted, whereas the total methane volume (V_CH4_) was calculated following the equation presented below.

% CH_4(dry)_ = % CH_4(wet)_ × (1 − P_vap_/P_amb_ + P_headspace_) (2)

V_CH4_ = (V_headspace_ T_STP_/T + V_biogas_) × % CH_4(dry)_ (3)

#### 3.4.3. Theoretical Biochemical Methane Potential

The empirical formula of the substrate used in the anaerobic digestion process was obtained based on its organic composition (C_n_H_a_O_b_N_c_S_d_) [71]. Additionally, the theoretical biochemical methane potential (TBMP) was determined using the Buswell and Boyle equations presented below [71,72].

C_n_H_a_O_b_N_c_S_d_ + (n − a/4 − b/2 + 3c/4 + d/2) H_2_O → (n/2 + a/8 − b/4 − 3c/8 + d/4) CH_4_ + (n/2 − a/8 + b/4 + 3c/8 + d/4) CO_2_ + cNH_3_ + dH_2_S (4)

TBMP (L CH_4_/KgVS) = 22.4 × (n/2 + a/8 − b/4 − 3c/8 − d/4)/12n + a + 16b + 14c + 32d (5)

#### 3.4.4. Kinetic Models

A modified Gompertz model was used to obtain the kinetics of the process; the model assumes that methane production follows the microbial growth pattern. The equation below describes the modified Gompertz model [59], in which y(t) is the cumulative biomethane production, A is the biomethane production potential, μ_m_ is the maximum biomethane production rate, e is a mathematical constant, λ is the lag phase period expressed in days and t is the cumulative time for biogas production in days.

y(t) = A e [−e ((μ_m_^e^/A) (λ − t) + 1)] (6)

### 3.5. Analytical Models

#### 3.5.1. Total and Volatile Solids

Total and volatile solids were determined gravimetrically, using a procedure described by the National Renewable Energy Laboratory (NREL) [70], in which 100 mg of sample was dried in a Binder oven (BINDER, Tuttlingen, Germany) at 105 °C until a constant weight was reached to obtain the total solids. Then, to quantify the volatile solids, the dried sample was heated at 575 °C for 4 h to calcine the sample.

#### 3.5.2. Chemical Composition

Lignin-like material was obtained gravimetrically according to the NREL methodology [73]. The sample was digested for one hour using sulfuric acid at 72% *v*/*v*. Thereafter, the digested sample was diluted to 4% *v*/*v* and heated at 121 °C for one hour to complete a double digestion. The separation of the liquid and solid fractions was performed by filtration using a Gooch “M” crucible. The solid acid residue was considered a lignin-like compound. Carbohydrates were diluted and moved into the liquid phase, and their identification was carried out by High-Performance Liquid Chromatography (HPLC). An Agilent Metacarb column (87H 300 × 7.8 mm) was used as the stationary phase, and sulfuric acid (5 mM) as the mobile phase. The column and detector temperatures were set at 50 and 35 °C, respectively. Quantification was done by creating a calibration curve with glucose, xylose and fucose standards, with concentrations ranging from 0.625 to 2.5 mg/mL.

#### 3.5.3. Elemental Traceability Analysis

Inorganic elements were quantified before and after the oxidative pretreatment, as well as during the alginate extraction process. An Inductively Coupled Plasma Optical Emission Spectrometer (Optima 8000, PerkinElmer Inc., Waltham, MA, USA) was used for the analysis. The ground and dried samples (0.5 g) were digested with 7 mL of nitric acid [3].

#### 3.5.4. Fourier Transform Infrared Spectrometry (FT-IR)

Infrared spectra were obtained using a Bruker Tensor II FT-IR spectrophotometer (Milton, ON, Canada). The region used for the analysis ranged from 4000 to 500 cm^−1^, with a resolution of 4 cm^−1^ and 32 scans [5].

#### 3.5.5. Proton Nuclear Magnetic Resonance (^1^H-NMR)

Mannuronic (M) and guluronic (G) acid fractions in sodium alginate obtained from SB and TS were determined using proton nuclear magnetic resonance (^1^H-NMR); additionally, alginic acid from Sigma-Aldrich (St. Louis, MO, USA) was used as a reference compound to compare the composition of the alginate. The analysis was performed using a Bruker 400 UltraShield spectrometer (Bruker BioSpin GmbH, Rheinstetten, Germany) at 600 MHz with a magnetic field of 14.1 T. The spectrum was obtained at 25 °C with 32 scans and a relaxation time of one second. ^1^H-NMR analysis was performed using one representative sample (*n* = 1) of the reference compound (alginic acid) and the alginate from SB and TS.

Samples were prepared using the ASTM F2259 method [74], which involves the preparation of an alginate solution at 0.1% *w*/*v*; the solution was then hydrolyzed with HCl (1 M) by adjusting the pH to 5.6 and heated at 100 °C for one hour. After that, the pH was decreased to 3.8 and boiled for 30 min. Finally, the pH was increased to 7.5 using NaOH (1 M) and freeze-dried. The dried samples were transferred into NMR tubes for dilution with deuterated water (99.9% *v*/*v*, Sigma-Aldrich, St. Louis, MO, USA) and stored at 4 °C for further analysis.

The equations used to calculate the mannuronic to guluronic acid ratio (M/G), in which F_G_ is the guluronic fraction, F_M_ is the mannuronic fraction, A_I_ is the area between 4.98 and 5.10 ppm, A_II_ is the area between 4.62 and 4.76 ppm and A_III_ is the area between 4.40 and 4.49 ppm, are presented below.

F_G_ = A_I_/A_II_ + A_III_ (7)

F_M_ = 1 − F_G_ (8)

M/G = F_M_/F_G_ = (1 − F_G_)/F_G_ (9)

### 3.6. Statistical Analysis

All analyses were performed in triplicate. Averages and standard deviations were calculated using STATISTICA 10.0 software (StatSoft, Inc., Tulsa, OK, USA); statistical differences were determined using the Tukey test (*p* < 0.05) with the same software. The graphs presented in this work were obtained using Origin software (Version 10.1, OriginLab Corporation, Northampton, MA, USA) and magnetic resonance spectra were analyzed using MestReNova software (Version 15.1.0, Mestrelab Research S.L., Santiago de Compostela, Spain) [75].

## 4. Conclusions

The oxidative pretreatment improves valorization by modifying the structure of the biomass and reducing the metal concentration; alginates obtained from *Sargassum* presented a low M/G ratio that makes it suitable for biomedical and environmental applications. In an integrated pathway, the waste from the alginate extraction process was used to produce biomethane, showing an increase in the yield and quality, making it a promising feedstock for biofuel production. Although the solid waste (W) was successfully valorized, it is important to consider the liquid streams generated during the process; further studies should focus on optimizing the oxidative pretreatment to reduce the volumes of remaining streams and evaluate their potential for reuse.

This study is the first step towards a zero-waste approach that promotes the integral valorization of *Sargassum*, turning the socioeconomical and environmental crisis into an opportunity to develop a circular economy in the Caribbean.

## 5. Patents

The oxidative pretreatment presented in this study has been protected through a Mexican patent application MX/a/2023/014346 entitled “Process for the Delignification and Reduction of Metal Content in *Sargassum* spp. through an Oxidative Treatment”, filed on 30 November 2023.

## Figures and Tables

**Table 1:** Composition of *Sargassum* biomass and the treated *Sargassum* on a dry basis.

Parameter (%)	*Sargassum* Biomass (SB)	Treated *Sargassum* (TS)
Ash	16.36 ± 0.21	16.31 ± 0.36
Lignin-like material	26.43 ± 1.75	7.60 ± 0.70
Glucose	6.05 ± 0.03	8.96 ± 0.20
Xylose	1.71 ± 0.01	3.10 ± 0.30
Fucose	1.10 ± 0.31	2.34 ± 0.20
C	37.62 ± 0.40	37.97 ± 2.47
H	4.04 ± 0.12	4.92 ± 0.20
N	0.98 ± 0.20	1.76 ± 0.30
S	0.78 ± 0.03	0.97 ± 0.10

**Table 2:** Mannuronic and guluronic acid fractions in *Sargassum* biomass (SB), treated *Sargassum* (TS) and the reference compound (alginic acid).

Sample	Alginate from SB	Alginate from TS	Alginic Acid
Guluronic acid fraction (F_G_)	0.62	0.75	0.48
Mannuronic acid fraction (F_M_)	0.38	0.25	0.52
M/G ratio	0.61	0.33	1.08

**Table 3:** Empirical formula from the feedstock, theoretical biomethane potential (TBMP), kinetic parameters obtained from the modified Gompertz model and biodegradability index (BI).

Sample	Empirical Formula	TBMP (mL CH_4_/gVS)	A (mL CH_4_/gVS)	λ (Days)	µ (mL CH_4_/gVS d)	R^2^	BI (%)
Blk	−	−	52.61 ± 2.41 ^a^	2.40 ± 034 ^f^	5.31 ± 0.17 ^g^	0.993	−
TSa	C_3.25_H_4.84_O_2.34_N_0.12_S_0.03_ + 0.97H_2_O → 1.60CH_4_ + 1.66CO_2_ + 0.12NH_3_ + 0.03H_2_S	425.07	149.89 ± 5.48 ^b^	2.58 ± 0.26 ^f^	15.01 ± 0.36 ^h^	0.996	29
TSb			135.20 ± 1.90 ^c^	3.82 ± 0.28 ^g^	32.28 ± 1.57 ^i^	0.998	31
TSc			129.40 ± 1.31 ^c^	4.29 ± 0.16 ^h^	24.67 ± 0.59 ^j^	0.993	30
Wa	C_3_H_4_O_1.71_N_0.06_S_0.02_ + 1.03H_2_O → 1.68CH_4_ + 1.37CO_2_ + 0.06NH_3_ + 0.02H_2_S	535.86	332.96 ± 4.09 ^d^	2.35 ± 0.21 ^f^	57.23 ± 1.61 ^k^	0.997	61
Wb			330.57 ± 5.18 ^d^	2.46 ± 0.21 ^f^	48.52 ± 1.28 ^l^	0.996	59
Wc			299.35 ± 11.09 ^e^	2.43 ± 0.38 ^f^	36.58 ± 1.53 ^m^	0.991	52

The different I:S ratios are represented next to the sample code as a = 2:1, b = 1:1 and c = 1:2; Blk = blank; TS = treated *Sargassum*; W = waste from the alginate extraction using treated *Sargassum*; VS = volatile solids; A = maximum production potential; λ = lag phase; µ = maximum production rate; R^2^ = coefficient of determination. Different letters indicate statistical differences (*p* < 0.05).

## Data Availability

The data presented in this study are available on request from the corresponding author. Restrictions apply to the availability of some data due to intellectual property protection associated with a patent application.
